# Extreme ST-segment elevations in seemingly no significant angiographic coronary artery abnormalities: a case report

**DOI:** 10.1186/s12872-019-1010-x

**Published:** 2019-01-29

**Authors:** M. Piels, T. Faes, J. Vainer

**Affiliations:** 1 Maastricht University Medical Center (MUMC+), P. Debyelaan 25, 6229HX, Maastricht, The Netherlands; 2 Gouverneur G. Ruijs de Beerenbroucklaan 36, 6123 AC, Holtum, The Netherlands

**Keywords:** MINOCA, STEMI, Tombstone ST elevation, Coronary spasm, Case report

## Abstract

**Background:**

Obstructive coronary artery disease is found in approximately 97% of patients presenting with ST-elevation myocardial infarction and 92% of patients with non ST-elevation myocardial infarction (Bainey KR, Welsh RC, Alemayehu W, Westerhout CM, Traboulsi D, Anderson T, et al. Int J Cardiol 264: 12–17, 2018). Recent studies showed that myocardial infarction without obstructive coronary atherosclerosis (MINOCA) is also associated with a long-term risk of adverse events (Bainey KR, Welsh RC, Alemayehu W, Westerhout CM, Traboulsi D, Anderson T, et al. Int J Cardiol 264: 12–17, 2018).. The following case illustrates that MINOCA may also be associated with short term adverse events (depending on the underlying mechanism).

**Case presentation:**

A 49-year old Caucasian male with no significant medical history was referred to our cardiac emergency department with acute chest pain. The ambulance ECG showed extreme ST-segment elevation anterolateral (‘tombstone sign’), which had resolved completely at arrival in the hospital. Coronary angiography showed no obstructive coronary artery disease. Conservative (medical) therapy was started and patient was discharged. Two days later he presented with recurrent cardiac ischemia with ventricular fibrillation. Coronary angiography showed no changes compared with earlier presentation. During admission to the ICU his clinical condition gradually deteriorated, eventually leading to his death. Post-mortem studies showed no significant atherosclerotic lesions. Massive myocardial infarction was found, probably caused by temporary occlusion of the left main coronary artery.

**Conclusions:**

Several pathophysiological mechanisms are recognized in MINOCA, of which vasospasm is the most probable one in this case. MINOCA is associated with increased over-all mortality and risk of ventricular arrhythmias. Therefore, additional testing should be considered when there is no explanation for the mismatch between ST-elevations (STEMI) and (no significant) coronary abnormalities.

## Background

Obstructive coronary artery disease is found in approximately 97% of patients presenting with ST-elevation myocardial infarction and 92% of patients with non ST-elevation myocardial infarction [1]. Recent studies showed that myocardial infarction without obstructive coronary atherosclerosis (MINOCA) is also associated with a long-term risk of adverse events [1]. The following case illustrates that MINOCA may also be associated with short term adverse events and may warrant further patient investigation regarding the underlying mechanism.

## Case presentation

**Fig. 1:**
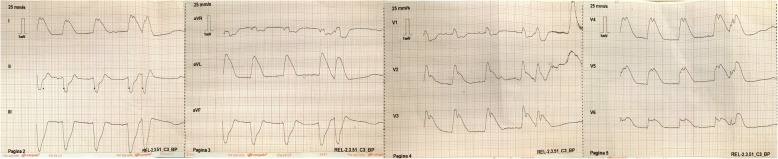
Ambulance recording of extreme ST-segment elevations at first presentation

**Fig. 2:**
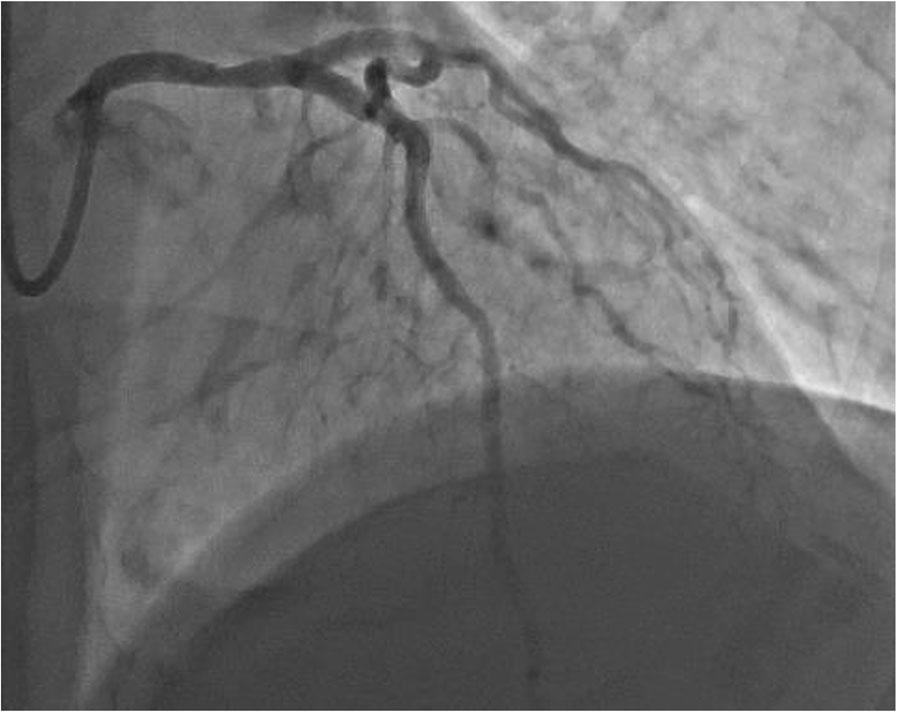
Coronary angiography of LMCA

A 49-year old Caucasian male with no significant medical history and no prior complaints of chest pain (smoking was his only known cardiovascular risk factor) was referred to our cardiac emergency department with acute chest pain during exertion. The ambulance ECG showed extreme ST-segment elevation anterolateral (‘tombstone elevations’), which had resolved completely at arrival in the hospital (Figs. 1 and 2).

**Fig. 3:**
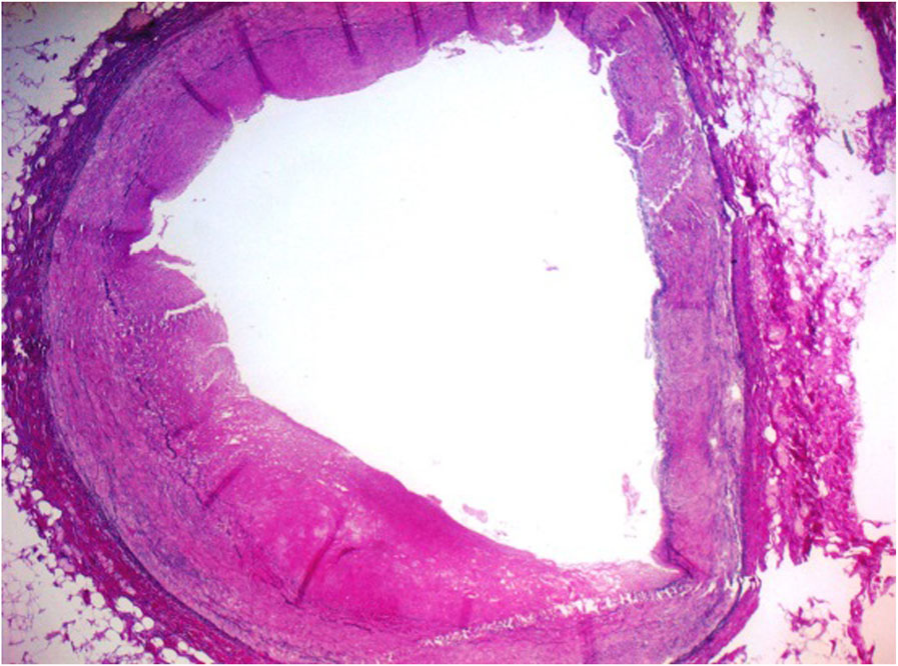
Microscopy showing 30–40% occlusion of the LMCA

In the hospital the patient immediately went to the catheterization laboratory for an emergency coronary angiography, which showed no significant lesions. At the bifurcation between LMCA and LAD wall irregularities were visible, possibly indicating either a small dissection or a passed thrombus (Fig. 3). Dual antiplatelet therapy was continued afterwards. During the next two days the patient did not have any complaints and no arrhythmias occurred. Ultrasonography showed no regional wall motion abnormalities, LV ejection fraction of 50% and no significant valvular disease. Patient received dual antiplatelet therapy, statins and ACE-inhibition. A beta blocker was also started, but had to be stopped due to symptomatic bradycardia. The patient was discharged 3 days after presentation. The out-patient follow-up visit was scheduled within 2 weeks after discharge. Unfortunately after discharge the patient resumed his smoking habits and refused to take any medication.

Two days later the patient presented at the emergency department after reanimation because of collapse due to ventricular fibrillation. Time of delay from onset until arrival of the ambulance was approximately 8 min. The ambulance ECG once again showed marked ST-elevations, which had resolved completely at hospital arrival. At arrival patient also had complete recovery of spontaneous circulation. An emergency coronary angiography was performed, which showed no changes compared to several days earlier and no clear cause of the VF. At first a conservative approach was chosen and the patient was admitted to the ICU. Intracoronary imaging (IVUS) of the LMCA was postponed awaiting neurological recovery.

After arriving at the ICU, the patient developed ventricular arrhythmias with loss of cardiac output. Attempts to restore output were not successful, which eventually led to implantation of veno-arterial extra corporal life support (ECLS). Repeated ultrasonography after placement of the ECLS showed worsening of the systolic LV-function (EF 10%), with only some wall movement in the RCA-fueled area. This was attributed to stunning post-reanimation and ECLS-implantation. During the next several days the systolic LV-function gradually improved to a point where the ECLS could be removed. In an attempt to prevent further ventricular arrhythmias and possible spasm, PCI of the angiographically not significant lesion of the LMCA was performed. Unfortunately, no neurological recovery occurred and the patient died 1 month later. The permission to perform an autopsy was granted (Table 1).

**Table 1:** Timeline

Days since admission	Time	Event
0	10:00	Presentation with STEMI
2		Discharge
4	08:35	Out of hospital cardiac arrest due to ventricular fibrillation
4	10:37	Emergency coronary angiography
4		Arrival on ICU: hemodynamically instable refractory ventricular tachycardia. Start extracorporeal life support
8		Stop extracorporeal life support
13		PCI LMCA – LAD
13–36		Long stay at ICU with clinical deterioration and no apparent neurological recovery
36	12:52	Patient died after treatment was stopped

### Post-mortem findings

The fore mentioned case left us wondering: what caused this man without any significant angiographic coronary lesions to present with such dramatic clinical consequences? Did we miss a significant lesion, did he have coronary spasms or is there another explanation?

Macroscopically the coronary arteries were open and showed no significant sclerotic lesions. The stent placed in the LMCA seemed to be open as well. On the Lactate dehydrogenase macroreaction (LDH) the entire left ventricle wall showed discoloration, with exception of the posterior wall. This finding indicates scarring and atrophy of the septum, anterior and lateral wall, indicating a massive myocardial infarction after occlusion of the LMCA. As the stent was not occluded the infarction probably occurred beforehand and was most likely the cause of the ventricular fibrillation at presentation.

**Fig. 4:**
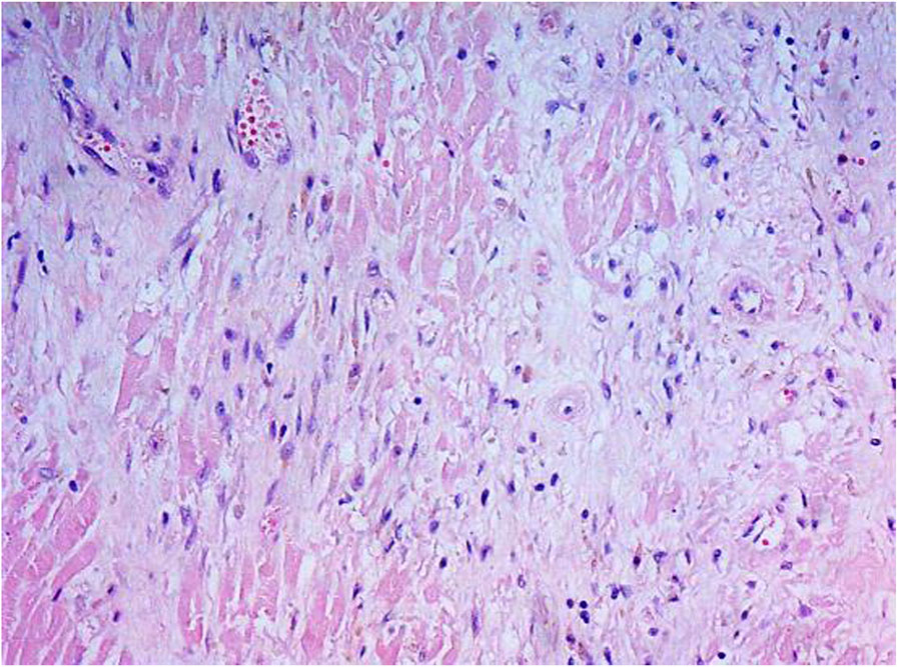
Microscopy showing presence of avital cardiomyocytes with fibroblast proliferation, macrophages and siderophages

**Fig. 5:**
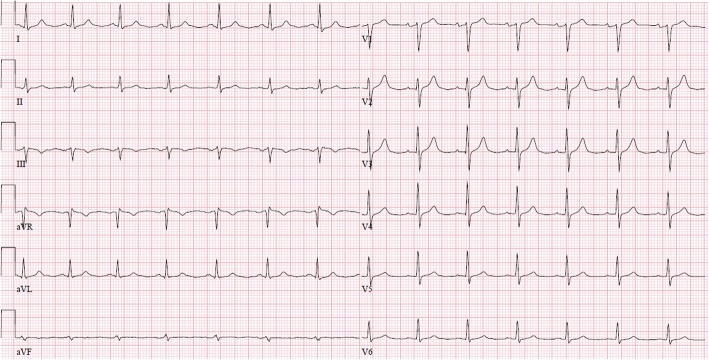
In-hospital recording of ECG at first presentation after resolution of ST-segment elevations

Microscopical examination of the heart showed extensive transmural infarction throughout the entire left ventricle, with avital cardiomyocytes, macrophages, siderophages, and fibroblast proliferation as a sign of starting fibrosis (Fig. 4). These findings are in agreement with a myocardial infarction which occurred several weeks ago (due to ischemia either during reanimation or before). The right ventricle only showed some focal ischemic changes with zones of vital myocardium. Detailed examination of the LMCA in the stented region showed a stable atheromatous plaque with 30–40% occlusion of the coronary artery and indentations in the wall, attributable to the stent (Fig. 5). The other coronary arteries, aorta and carotid arteries only showed minor atherosclerotic changes without significant occlusions.

## Discussion and conclusions

This case is an extreme example of MINOCA (Myocardial Infarction with No Obstructive Coronary Atherosclerosis). Post-mortem findings showed clear signs of atherosclerotic changes in the LMCA and extensive infarction, where no significant angiographic lesions were visible. There were no signs of myocarditis or other explanations found for the rapid deterioration of the patient. Several pathophysiological mechanisms are recognized in MINOCA [2], of which vasospasm is the most probable one in this case.

Coronary spasms might be an explanation for this extreme presentation, as showed by previous case reports showing large infarctions due to spasms of the LMCA [3, 4]. The extreme elevations in our case could well fit spasms of either the LMCA or of both LAD and RCx. Further functional testing (such as spasm provocation testing) might have revealed additional information earlier on in this case. These tests however are not (and should not be) standardly performed in every patient. In patients presenting with extreme ECG or biochemical abnormalities without clear angiographic abnormalities though, these tests could be considered more readily.

Rupture of an eccentric plaque might be another possible explanation for MINOCA, although post-mortem findings do not support this theory in our patient. While angiographically no significant abnormalities were seen, postmortem findings did show an occlusion of 30–40% of the LMCA after stenting.

While the majority of patients presenting with a myocardial infarction has significant coronary artery disease, a significant proportion of patients shows no significant coronary abnormalities (a prevalence of 8.8% in NSTEMI is reported [2] and approximately 3% in STEMI [5]). One study in patients with MINOCA showed an increased prevalence of ventricular arrhythmia of 13.8% during hospitalization [6], especially in patients presenting with ST-segment elevation and patient who had transmural late gadolinium enhancement on CMR. Furthermore, non-obstructive coronary artery disease is associated with increased overall 1-year mortality (though mostly driven by greater non cardiac mortality) [5].

In conclusion myocardial infarction without obstructive coronary atherosclerosis is associated with increased over-all mortality and risk of ventricular arrhythmias. Therefore, additional testing should be considered when there is no satisfying explanation for the mismatch between ST-elevations (STEMI) and (no significant) coronary abnormalities.

## Abbreviations

ACE: Angiotensin converting enzyme
CMR: Cardiac magnetic resonance imaging
ECG: Electrocardiogram
ECLS: Extra corporal life support
EF: Ejection fraction
FFR: Fractional flow reserve
ICU: Intensive care unit
IFR: Instantaneous wave-free ratio
IVUS: Intravascular ultrasound
LAD: Left anterior descending coronary artery
LDH: Lactate dehydrogenase
LMCA: Left main coronary artery
LV: Left ventricle
MINOCA: Myocardial infarction without obstructive coronary atherosclerosis
NSTEMI: Non ST-elevation myocardial infarction
PCI: Percutaneous coronary intervention
RCA: Right coronary artery
STEMI: ST-elevation myocardial infarction
VF: Ventricular fibrillation
