# Correction to: Extreme ST-segment elevations in seemingly no significant angiographic coronary artery abnormalities: a case report

**DOI:** 10.1186/s12872-019-1020-8

**Published:** 2019-03-26

**Authors:** M. Piels, T. Faes, J. Vainer

**Affiliations:** 1 Maastricht University Medical Center (MUMC+), P. Debyelaan 25, 6229HX Maastricht, The Netherlands; 2 Gouverneur G. Ruijs de Beerenbroucklaan 36, 6123 AC Holtum, The Netherlands

**Correction to: BMC Cardiovasc Disord (2019) 19:28**

**https://doi.org/10.1186/s12872-019-1010-x**

Following the publication of the original article [1], the author reported several figures have the wrong number related to them. Figure 2 should be 3, figure 5 should be 2, figure 3 should be 5.

It has been corrected in the original article as well.

The publisher apologizes for any inconvenience caused by this error.
